# 'You had to do something': prescribing antibiotics in Scotland during the COVID-19 pandemic restrictions and remobilisation

**DOI:** 10.1038/s41415-021-3621-8

**Published:** 2021-11-23

**Authors:** Eilidh M. Duncan, Beatriz Goulao, Janet Clarkson, Linda Young, Craig R. Ramsay

**Affiliations:** 41415111951001 Health Services Research Unit, Institute of Applied Health Sciences, University of Aberdeen, Aberdeen, UK; 41415111951002 NHS Education for Scotland, Edinburgh, UK; Dental Health Services Research Unit, School of Dentistry, University of Dundee, Dundee, UK; 41415111951003 NHS Education for Scotland, Edinburgh, UK

## Abstract

**Introduction **The COVID-19 pandemic brought about seismic change for dentistry including the direction to provide remote advice and prescribe analgesia and antimicrobials. The possibilities for care have widened, but the impact of both restrictions and remobilisation on antibiotic prescribing is not known.

**Aims **To report the impact of COVID-19 restrictions and remobilisation on dental antibiotic prescriptions and explore dentists' intentions and attitudes towards antibiotic prescribing.

**Design and setting **Public Health Scotland national prescribing and claims data are reported alongside an online survey of Scottish general and public health service dentists including closed and open-ended questions.

**Results **Antibiotic prescribing rose by 49% following the suspension of routine dental care, to a peak of 34,993 antibiotics (July 2020). The data also show that since the remobilisation of NHS dental care, antibiotic prescribing remains raised at levels around 28% higher than pre-pandemic. The survey highlights dentists' frustrations and concerns about this increased use of antibiotics. Most dentists intend to reduce their prescribing; however, significant challenges to this being realised were raised.

**Conclusions **The previous success within dentistry to protect against the development of antimicrobial resistance has suffered a knock-back during the pandemic. A renewed focus on reducing unnecessary antibiotics within dentistry is required but, crucially, needs to be approached sensitively alongside the current backdrop of challenges within the service.

**Supplementary Information:**

Zusatzmaterial online: Zu diesem Beitrag sind unter 10.1038/s41415-021-3621-8 für autorisierte Leser zusätzliche Dateien abrufbar.

## Key points

- Highlights the increased antibiotic prescribing that resulted during the COVID-19 pandemic using routine national prescribing and claims data.
- Highlights that antibiotic use remains at a higher level despite the remobilisation of NHS dentistry.
- Details the results of a national survey of Scottish general and public dentists reporting ongoing challenges.
- Reports that dentists are aware they are prescribing at higher levels, are frustrated and concerned by this, and wish to reduce this but are facing challenges to do so.

**Supplementary Information:**

Zusatzmaterial online: Zu diesem Beitrag sind unter 10.1038/s41415-021-3621-8 für autorisierte Leser zusätzliche Dateien abrufbar.

## Introduction

Antimicrobial resistance is a growing and significant global threat that has wide-ranging impacts on health, finances, food sustainability, security, environmental wellbeing and socioeconomic development.^1^ Antibiotic use in human medicine plays a key role in the development and spread of antimicrobial resistance^2^ and the optimisation of antibiotic use is a primary focus of the UK's 2019-2024 national action plan to tackle antimicrobial resistance.^1^

The efforts to control antimicrobial resistance through reducing the unnecessary exposure to antibiotics have seen some success in recent years. Total antibiotic use in Scotland decreased by 7.6% from 2015-2019, and over the same time period, the use in primary care decreased by 9.1% to the lowest figure since 1993.^3^ Within dentistry specifically, the rate of antibiotic use reduced by 17.7% (2015-2019) and accounted for around 7% of the total antibiotic use in primary care in Scotland 2018-2019.^3^ Dental pain and infection can be effectively managed through physical dental interventions; however, this requires face-to-face dental care which was constrained during COVID-19. There is emerging evidence to suggest that the pandemic has had an impact on antibiotic prescribing. A recent study^4^ highlighted that in the early months of the pandemic in England (April-July 2020), dental antibiotic prescribing was 25% higher compared with the same months in 2019.

The early months of the COVID-19 pandemic brought severe curtailment to the delivery of dental care within the UK. In Scotland, the Chief Dental Officer (CDO) wrote to all practices announcing the suspension of routine dental care on 23 March 2020^5^ and NHS England announced the suspension and deferment of all routine, non-urgent dental care on 25 March 2020.^6^ During the suspension of routine dental care, Scottish practices were instructed to operate on an 'advice, analgesia, anti-microbial' basis with the rationale stated as 'where an extraction is not indicated an anti-microbial will provide relief until the tooth can be opened and drained by an urgent dental care team'.^5^ Patients with dental care concerns were remotely triaged by practices and those with an urgent need that could not wait were directed to the designated urgent dental care centre for the area. In England, the Office of the Chief Dental Officer (CDO) produced similar advice: dentists should not provide routine care; should offer telephone triage and advice, giving prescriptions where necessary; and should refer patients requiring active emergency treatment to regional urgent care centres. These arrangements stayed in place during the three-phase remobilisation of NHS dental services, with a return to emergency-only General Dental Services care in Scotland from 22 June 2020, routine non-aerosol generating procedures (AGPs) returning from 13 July 2020 and AGPs for urgent dental care only returning from 17 August 2020.^7^ The full range of NHS dental treatments (including AGPs for routine care) could not be provided until November 2020.^8^ On 21 June 2021, the CDO for Scotland wrote to the profession in response to recommendations raised by the Scottish Antibiotic Prescribing Group to emphasise that the first-line management of acute dental conditions should revert to clinical management rather than antibiotic prescribing.^9^

It is not currently known what the impact of COVID-19 and the remobilisation of NHS dentistry has had on the levels of antibiotic prescribing, nor is it known whether the increased dental antibiotic prescribing figures seen in England is reflected in the data for the rest of the UK. Furthermore, the prescribing data only provide part of the picture and there is a need to hear from dentists themselves about the experiences and feelings that lie behind the antibiotic prescribing figures. This paper reports on an exploration of the Scottish data to report the impact of COVID-19 and remobilisation on dental antibiotic prescriptions, and the results of a national questionnaire of General Dental Services and Public Dental Services dentists in Scotland exploring dentists' intentions and attitudes towards antibiotic prescribing.

## Methods

### Prescribing and treatment data

We used Prescribing Information System for Scotland (PRISMS) data to obtain overall NHS antibiotic prescriptions and Management Information Dental Accounting System (MIDAS) data to obtain the total number of NHS claims. Both databases are held by Public Health Scotland. Data in PRISMS are identified by list number, which is a unique identifier of a dentist in a given dental practice. Antibiotic prescription data comprise all antibiotic items (BNF section 5.1 'antibacterial drugs') prescribed by primary care dentists in Scotland and dispensed in community pharmacy. We calculated percentages of change between pre-pandemic levels of antibiotic prescription (January 2019) and post-pandemic levels by using a standard proportion calculation; that is, (prescriptions [post-pandemic] minus prescriptions [pre-pandemic] divided by prescriptions [pre-pandemic]). Claims data comprise course of treatment claims and non-course of treatment claims; for example, patient registrations. Data were included from January 2019 to May 2021. Prescribing rates were calculated as the total national number of antibiotic items prescribed by primary care dentists in Scotland per month divided by the total national number of claims in Scotland in the same month and multiplied by 100. They are presented with descriptive statistics - average (mean), standard deviation (SD) and range (minimum-maximum).

### Survey development

Ethical review of the survey was completed by University of Aberdeen Research Governance on 30 September 2020 which classed this project as service evaluation. The project was registered with the Quality Improvement and Assurance Team (Project ID 5196) on 7 December 2020.

The questionnaire included both open- and closed-ended questions. Items in the questionnaire were developed based on previous qualitative research exploring dental antibiotic prescribing.^10^^,^^11^ The questionnaire was piloted with six dentists (invited from known networks including the Scottish Dental Practice Based Research Network) who provided feedback to improve clarity of questions and to ensure response options were appropriate for both General Dental Services (GDS) and Public Dental Services (PDS) respondents. Within the questionnaire, the term 'lockdown' was defined as the period of March 2020 when routine dental care was suspended.

### Procedure

E-mails inviting dentists to complete the survey were sent via the NHS Education for Scotland Portal on 8 December 2020. One reminder e-mail was sent on 7 January 2021.

### Sample size

To achieve a precision of 5% with over 95% power to estimate the proportion of dentists that have 1) increased their prescribing during the pandemic or 2) intend to decrease their prescribing in the future, we required 300 responses. This assumes a population of 4,500 dentists in Scotland and an expected prevalence of 80% or above for each outcome. This calculation was done using OpenEpi, version 3, open source calculator (www.openepi.com/).

### Analysis

Closed questions are presented with the appropriate descriptive statistics. When percentages are presented, they are calculated using the total number of responders as the denominator. Open-ended questions were extracted verbatim into Excel and thematic analysis was conducted by reading and re-reading the comments and iteratively developing a coding frame.

## Results

### National antibiotic prescribing data

#### Description of the dataset

There were 8,097 list numbers with any prescribing information in PRISMS in the period between January 2019 and May 2021.

#### Antibiotics prescribed in Scotland 2019-2021

**Fig. 1:**
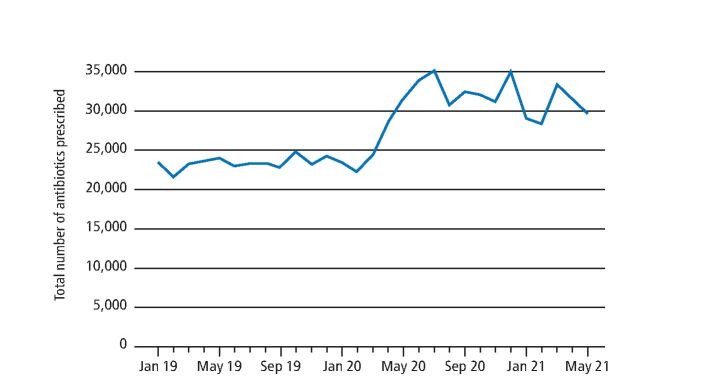
Total number of antibiotic items prescribed in Scotland

Figure 1 shows the total number of antibiotic items prescribed in Scotland from January 2019 until May 2021. In January 2019 (pre-pandemic), 23,445 antibiotics were prescribed. During the peak of the pandemic (July 2020), the total went up by 49%, when compared with pre-pandemic, to 34,993 antibiotics. Even though that maximum has not been reached again, prescriptions remain consistently above 25,000 antibiotics per month, with the last month of data available at time of publication (May 2021) registering antibiotic prescriptions just below 30,000 (around 28% higher than pre-pandemic levels).

This increase in the number of antibiotics prescribed comes at the same time as overall reduced dental treatment activity, as measured by the number of claims of treatment in Scotland. Prescribing rates (the number of antibiotics prescribed per 100 claims of treatment) highlight the proportion of patients being treated who were prescribed antibiotics. The average monthly rate of antibiotic prescriptions per 100 claims pre-pandemic (January 2019-March 2020) was 5.4 antibiotics per 100 claims (SD for national rates corresponding to these 15 months is 1.5). This increased markedly to an average monthly rate of 148.7 antibiotics per 100 claims (SD = 143.7, range = 21.0-364.6) during the period of most intense restrictions to service (April-August 2020). Rates then reduced to 17.7 antibiotics per 100 claims (SD = 1.9, range = 16.4-19.9) during the remobilisation period (September-November 2020). However, data for the period December 2020-May 2021 highlight that prescribing rates have not reduced to pre-pandemic levels, with an average of 17.3 antibiotics per 100 claims (SD = 3.9, range = 13.8-23.3).

### National dental questionnaire

#### Responses and practice context

Responses were obtained from 311 dentists, of whom 278 (89%) were from GDS and 32 (10%) were working in PDS (one respondent did not declare). All health boards within Scotland except Shetland returned at least one response (see online supplementary information) and the distribution of dentists across health boards in our sample is similar to the distribution of GDS dentists in Scotland. The majority of respondents provided NHS dental services for the whole, main or equal proportion of their practice time (n = 267), with 44 providing whole or mostly private provision.

#### Reflections on the suspension and restrictions placed on NHS dentistry during the pandemic

Frustration was expressed in the open responses about the restrictions, guidance and policies brought into effect during the pandemic: 'there was no good reason for practices to be closed'* (*participant 191); 'we should never have prevented dental services' (participant 246); 'practices should never have been told to close and extractions should have continued throughout the pandemic. I am still unaware of any evidence to show this would have been unsafe' (participant 307).

Harms caused by the impact of the restrictions on treatments were raised, including 'a loss of satisfaction and trust in dental care for these patients' (participant 143); 'the huge backlog of patients, the extreme reduction in capacity within the GDS [...] severe deterioration in the nation's dental health and an uptick in patients attending emergency departments and their GPs due to completely unavoidable lack of access to their dentist. Complaints are going to skyrocket' (participant 190).

Deterioration to patients' dental health was mentioned as a concern alongside frustration about being prevented from effectively helping patients in need: 'it was wrong [prescribing antibiotics for pulpits] and felt wrong, it was a bad time not being able to help people in pain' (participant 246).

Comments also suggested a loss of trust in regulatory bodies and in guidance, and a loss of professional autonomy: 'we are health professionals who have been treated like little children. I feel strongly about this. A wholly negative experience' (participant 209).

#### Perceptions about changes in antibiotic prescribing during the pandemic

Links were made by respondents between the changes to delivery of dental care and an increased use of antibiotics: 'first we are to reduce prescribing and then we are prevented from looking after our patients and asked to hand them out like sweeties' (participant 209); 'antibiotics should never have been used as a substitute for treatment [...] it should not have been forced upon us during lockdown' (participant 219). Respondents commented on their feelings that antibiotic prescribing had increased during COVID-19: 'I was shocked by the amount of antibiotics I prescribed over lockdown. All necessary because I couldn't see the patients face-to-face to treat them properly' (participant 100).

**Fig. 2:**
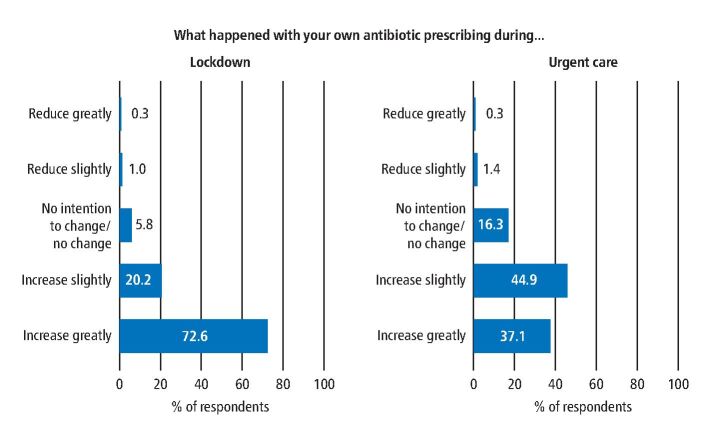
Perceptions about change in antibiotic prescribing during pandemic restrictions

The vast majority of respondents reported they had increased their antibiotic prescription slightly or greatly during the suspension of face-to-face care in March 2020 (92%, n = 271) and the emergency-only GDS care in June 2020 (82%, n = 241) compared to pre-COVID, as shown in Figure 2.

Comments were also made about prescribing antibiotics counter to clinical assessment of need during COVID-19 to permit patients to access urgent treatment: 'urgent dental care centres during lockdown were declining patients if they haven't taken at least one course of antibiotics. You can understand how ridiculous this looks to patients' and dentists' eyes' (participant 272). This happened even when their clinical judgement was that it would not be of benefit: 'we were given no option but to Px [prescribe] ABs [antibiotics] even when we knew it would do no good' (participant 314). These comments are reflected in the quantitative responses where 84% (n = 262) of respondents were unsure, disagreed, or strongly disagreed that prescribing antibiotics when no other treatment is possible will effectively solve or manage the patient's condition. Most respondents (82%, n = 256) agreed or strongly agreed that prescribing antibiotics when no other treatment is possible goes against their views of best practice (Table 1).

**Table 1:** Quantitative questionnaire items related to attitudes to antibiotic prescribing (N = 311)

	Strongly disagree	Disagree	Neither agree/disagree	Agree	Strongly agree	Not answered
How strongly do you agree or disagree that prescribing antibiotics when no other treatment is possible…						
Ensures patient safety when no AGPs are available	22 (7%)	63 (20%)	67 (22%)	128 (41%)	21 (7%)	10 (3%)
Can cause patients harm	2 (1%)	17 (6%)	70 (23%)	156 (50%)	56 (18%)	10 (3%)
Will effectively solve or manage patients' condition	68 (22%)	113 (36%)	81 (26%)	35 (11%)	4 (1%)	10 (3%)
Will negatively impact patients' dental health	8 (3%)	54 (17%)	108 (35%)	93 (30%)	38 (12%)	10 (3%)
Goes against my views of best practice	2 (1%)	13 (4%)	29 (9%)	132 (42%)	124 (40%)	11 (4%)
Goes against guidance	7 (2%)	27 (9%)	55 (18%)	108 (35%)	102 (33%)	12 (4%)
Preserves a good patient/dentist relationship	14 (5%)	44 (15%)	92 (31%)	132 (44%)	19 (5%)	10 (3%)
How strongly do you agree or disagree…						
That not providing antibiotics is likely to cause conflict with my patients	9 (3%)	47 (15%)	85 (27%)	129 (42%)	30 (10%)	11 (4%)
Misuse of antibiotics is not a problem in dentistry	91 (30%)	154 (30%)	29 (9%)	16 (5%)	9 (3%)	12 (4%)
Responsible use of antibiotics is important within dentistry	4 (1%)	2 (1%)	6 (2%)	105 (34%)	183 (59%)	11 (4%)

Reports were also shared of prescribing antibiotics as a means of providing some level of care when nothing else was possible: 'Antibiotics were often prescribed in lockdown when the symptoms were not indicative that antibiotics would have any effect just because "you had to do something"' (participant 57).

There were strong feelings expressed such as: 'I felt terrible giving patients antibiotics that I knew were unlikely to help' (participant 311); 'the way antibiotics was [sic] prescribed by the hubs (and some GDPs) during lockdown was verging on criminal' (participant 287); and 'patients were having 5+ courses of antibiotics as no access to dentistry. Totally disgraceful' (participant 294).

Further quantitative responses related to attitudes to prescribing antibiotics are presented in Table 1.

#### The future for antibiotic prescribing

Some of the respondents reflected that antibiotic prescribing was already returning to a reduced pre-COVID-19 level: 'during lockdown was difficult [...] prescribing them [antibiotics] for 3 months when I knew this was not the ideal treatment was counter productive and I am very happy not to be in that situation anymore' (participant 49); 'Luckily being able to carry out treatments has ensured a decrease in prescribing already' (participant 279).

However, for others, the picture has not yet changed and antibiotic prescribing remains at a higher level: 'I think we can't expect antibiotic prescription to reduce until we are working with less restrictions' (participant 275); 'how can we reduce the antibiotic prescribing with only enough PPE and time to see 5 AGPs a day if patients are in pain?' (participant 84). As well as the restrictions on AGP appointments, PPE and time, references were also made to the impact of the backlog and increased waiting lists, and the lack of dentists with face-fitted masks.

When asked to quantitatively rate their intentions and expectations about antibiotic prescribing, the vast majority of respondents (80%) intend to decrease their prescriptions and 80% expect antibiotic prescribing to reduce in dentistry overall.

#### The role of patients in antibiotic prescribing

Concerns were expressed by respondents that patients will have greater expectations for antibiotics post-COVID-19: 'Many patients are now happier to have antibiotics over treatment and expect things to be as they were in lockdown' (participant 183); 'Over the last number of years I feel the profession on the whole has been trying to reduce antibiotic prescriptions and instead using local measures to deal with the cause of the infection. We have been slowly changing public perception that antibiotics aren't always necessary or indicated and they're slowly accepting this. During COVID we have done the exact opposite of this and have only been prescribing antibiotics in a lot of cases and have undone years of hard work. I feel this has set us back a lot in terms of patient expectation' (participant 329). Furthermore, 42% (n = 132) of respondents agreed that prescribing antibiotics when no other treatment is possible preserves a good patient/dentist relationship. Around the same proportion (42%; n = 129) agreed that not providing antibiotics is likely to cause conflict (Table 1).

Comments also described the role of patients in terms of directly requesting antibiotics and refusing treatments so that antibiotics are the only route left: 'when a patient doesn't want a tooth out but they can't have rct [root canal treatment] for a weeks [sic] what other option is there?' (participant 316); 'patients think antibiotics are the golden nugget that will fix their pain and don't understand it will only temporarily take down the infection. Pts [patients] think Abs [antibiotics] will solve all their emergency issues and ask blatantly for them. Patients have boxes of Abs [antibiotics] lying in drawers at home [...] It's an absolute wild west out there when it comes to antibiotics' (participant 255).

#### The magnitude of the problem and the impact of dentistry

The vast majority of participants (93%, n = 288) agreed or strongly agreed that responsible use of antibiotics is important within dentistry (Table 1). Only a relatively small proportion (17%, n = 54) agreed that antibiotics misuse is not a problem in dentistry (Table 1). In the open comments, some respondents did take the opportunity to raise queries about the true impact dental antibiotic prescribing plays in the overall picture of antimicrobial resistance development: 'dentistry is a drop in the ocean compared to decades of misuse in the medical profession' (participant 278); 'their [antibiotics] use in dentistry is justifiable' (participant 37). However, this was balanced against others' comments acknowledging the part dentistry has to play in decreasing unnecessary antibiotic prescriptions and that over-use is not a uniquely COVID-19-related phenomenon.

## Discussion

This paper combines data from mixed sources to provide a unique insight into how the pandemic has impacted dentists' prescribing behaviour, perceptions and experiences. The data reported in this study from the national routine prescribing datasets show that dental antibiotic prescribing rose significantly in Scotland during the COVID-19 pandemic, echoing the picture reported during the early months of the pandemic in England.^4^ This study also provides further insight that higher levels of prescribing persist even with the remobilisation of NHS treatment (June to November 2020). Dentists report feeling compelled to 'do something' for patients and that prescribing antibiotics satisfied this need to provide some level of care during the COVID-19 restrictions when treatment options were so limited.

Dentists are aware that they are prescribing antibiotics at higher levels and report feeling frustrated about prescribing antibiotics against their clinical judgement. They expressed concerns about the future impact of this increased antibiotic prescribing and most respondents reported an intention to reduce their antibiotic prescribing in the future. However, significant challenges were reported to reducing prescribing in the context of ongoing restrictions to the numbers of patients that can be seen and the large backlog of patients needing attention. The anxiety shared in this study, and others,^12^ around the anticipated backlog of treatments and patients needing care suggests that there is a clear need to help support practices to manage this alongside the remobilisation of care. Furthermore, there has, as yet, been no real change in how dentists can deliver care with social distancing measures, PPE and fallow time restrictions remaining in place.

Dentists expressed feeling a strong sense of duty to act rather than leave patients in pain waiting with no treatment. This resonates with previous work,^12^ highlighting that dentists would struggle with not providing a service to patients during the pandemic, seeing this as an integral part of their identity as a healthcare professional. Dentists in this study reported that prescribing antibiotics satisfied this 'need to do something', despite the fact antibiotics are unlikely to resolve much dental pain. This strong sense of duty to act may also be, in part, driven by concerns about the medico-legal consequences of withholding care. The potential legal consequences of not providing care when patients are clearly in need and may come to harm if not treated is a concern for UK dentists.^13^

Furthermore, going from the established guidance on management of acute dental problems to the advice, analgesia, antimicrobial (AAA) approach during the pandemic may also raise medico-legal concerns. Indeed, most dentists in this study agreed that prescribing antibiotics just because no other treatment is available goes against best practice. The move to AAA during the restrictions to dental services was not only applied within the UK but formed part of World Health Organisation global guidance.^14^ We would encourage the global dental community to reflect on lessons learned from this approach and come together to prepare for the next pandemic.

There are some strengths and limitations of this study to note. The data in this paper reflect the picture of antibiotic prescribing in Scotland only and generalisability beyond this context is uncertain. However, the use of routinely collected datasets to provide granular antibiotic prescribing data is a major strength which would not be possible in all countries. The claims data that have been used to calculate the proportions of patients being seen who received antibiotics may result in a degree of over-estimation of prescribing rates per claims, as submissions of claims may have been disrupted during the pandemic. Although the prescribing rates per claims figures should be interpreted with caution, the clear increase and sustained rise observed in the total number of antibiotic items comes at a time when the number of patients being seen was dramatically reduced.

The results reported in this paper suggest that a renewed focus on optimisation and reduction of antibiotic prescribing is warranted within dentistry but, crucially, this needs to be framed sensitively in what continues to be challenging times for dentistry. Alongside the frustrations expressed about the restrictions to service, loss of professional autonomy and loss of trust in regulatory bodies reported in this study, there is also the mental health impact which makes sensitive framing so critical. Recent research^15^ demonstrates the pandemic-related mental health burden within dentistry, with 27% of survey respondents reporting significant depressive symptoms and 55% reporting emotional exhaustion. The pressures and mental health impacts being faced within dentistry are likely to be a persisting challenge going forward, and renewed focus on mental health awareness and support within dentistry is apparent in recent initiatives.^16^^,^^17^

The dentists in this study also highlighted the influential role patients can play in antibiotic prescribing, resonating with previous work^11^ but also raising concerns about the potential for greater future demand following the widespread use of antibiotics during the pandemic. However, recent research^18^ examining Twitter communications about dental health and care during the pandemic suggested that the overuse of antibiotics and antimicrobial resistance was a concern to the general public too. These findings suggest that initiatives that target dentist-patient treatment discussions and decision-making may be indicated.

Most dentists in this study felt that reducing antibiotic prescribing is very important and recent exploration of the Public Health Scotland prescribing in the community datasets^19^ highlights the increasing impact dentistry has within primary care antibiotic prescribing. In the six months before the pandemic restrictions of March 2020, on average, 7% of the total primary care antibiotic prescribing was within dentistry; however, in the six months from April-September 2020, this had increased to 12%. This represents an increase from dentists prescribing 1 in every 14 antibiotics in primary care to prescribing 1 in every 9 antibiotics. This further supports a renewed focus on antibiotic optimisation within dentistry and there are successful evidence-based initiatives that have been shown to help. Providing dentists with personalised prescribing feedback reports, alongside a short actionable message based on behaviour change theory, successfully reduced antibiotic prescribing rates.^20^ A novel initiative to examine whether the addition of in-practice training support to these feedback reports is currently underway to see if this brings about further reductions in antibiotic prescribing.^21^ The in-practice training support element may help dental practices to find a way to move forward with reducing dental antibiotic prescriptions in the current and future post-COVID-19 dental care landscape.

## Conclusions

The previous success within dentistry to protect against the development of antimicrobial resistance has suffered a knock-back during the pandemic. Antibiotic prescribing rose significantly in dentistry during the COVID-19 pandemic and prescribing continues at a higher level than pre-pandemic. A renewed focus on reducing unnecessary antibiotics is required but dentists report ongoing challenges that may stymie prescribing levels falling. Efforts to help support reducing dental prescribing need renewed focus alongside careful tailoring and sensitive framing for what continues to be a difficult new landscape for dentistry.

## Supplementary Information

Supplementary Table 1 (PDF 727KB)
