# Supplementary material for: 'You had to do something': prescribing antibiotics in Scotland during the COVID-19 pandemic restrictions and remobilisation
Source: Br Dent J. 2021 Nov 23:1–6. Online ahead of print. doi: 10.1038/s41415-021-3621-8 (PMC8609985; doi:10.1038/s41415-021-3621-8)
Supplement: Supplementary file 1 — Supplementary Table 1 (PDF 727KB) [file 41415_2021_3621_MOESM1_ESM.pdf]

Supplementary Table 1: Distribution of responses by NHS health board region and distribution of GDS[a] dentists in Scotland

**Health Board**	**Number of Responses**	**Proportion of sample**	**Proportion of GDS[a] dentists in Scotland**
Greater Glasgow & Clyde	66	21.2	25.9
Lothian	64	20.6	17.4
Grampian	33	10.6	8.9
Tayside	28	9.0	7.9
Lanarkshire	25	8.0	12.9
Fife	25	8.0	6.4
Ayrshire & Arran	24	7.7	6.2
Highland	21	6.8	4.8
Forth Valley	7	2.3	5.0
Borders	6	1.9	1.7
Orkney	5	1.6	0.3
Dumfries & Galloway	4	1.3	2.5
Eileanan Siar Western Isles	1	0.3	0.1
Shetland	0	0	0.01
Not reported	2	0	
**Total**	**311**		

[a]GDS = General Dental Services
